# Seroprevalence of Hepatitis B Surface Antigen and Anti Hepatitis C Antibody in Zahedan City, Iran: A Population-Based Study

**DOI:** 10.5812/hepatmon.6618

**Published:** 2012-09-30

**Authors:** Alireza Ansari-Moghaddam, Mohammad Reza Ostovaneh, Batool Sharifi Mood, Esmail Sanei-Moghaddam, Amirhossein Modabbernia, Hossein Poustchi

**Affiliations:** 1 Health Promotion Research Center, Zahedan University of Medical Sciences, Zahedan, IR Iran; 2 Digestive Disease Research Institute, Shariati Hospital, Tehran University of Medical Sciences, Tehran, IR Iran

**Keywords:** Prevalence, Epidemiology, Hepatitis B Surface Antigens, Hepatitis C Antibodies, Risk Factors, Iran

## Abstract

**Background:**

There have been studies regarding the prevalence of hepatitis B surface antigen (HBsAg) and anti-hepatitis C antibody (HCVAb) in Iran. However, the majority of these have reported a variety of rates, depending on their study population, which limits the generalizability of their results to the general population. On the other hand, cultural diversity in the different provinces of Iran also necessitates the performing separate population-based studies in the various regions.

**Objectives:**

To evaluate the population-based prevalence of HBsAg and HCVAb and their correlates in Zahedan City, Iran.

**Patients and Methods:**

Included in this study were 2587 individuals, using a random and cluster sampling approach. The participants were drawn from the Family Registry of the public health centers in Zahedan City, Iran, from 2008 to 2009. Following data collection from the interviews, subjects were assessed for seropositivity of HBsAg and HCVAb. We then calculated the prevalence of HBsAg and HCVAb, and evaluated these viral markers for an association with; age, sex and potential risk factors.

**Results:**

Weighted seroprevalence of HBsAg and HCVAb was 2.5% (CI 95% : 1.9 to 3.3 %) and 0.5% (CI 95% : 0.27 to 0.9 %), respectively. Prevalence of HBsAg increased significantly with age (P value < 0.001), but this was not true for HCVAb (P value: 0.67). We observed no sex dominance in the prevalence of HBsAg (3.2% and 2.2% for males and females, respectively, P value: 0.15) or HCVAb (0.4% and 0.7% for males and females, respectively, P value: 0.27). In a multivariate regression analysis, every additional year in age resulted in a 2% increment in the odds of HBsAg seropositivity. HBsAg was also three times more prevalent among married, than single subjects (with a P value reaching toward significance: 0.065) in multivariate analysis. Prevalence of HCVAb did not differ with respect to any of the potential risk factors.

**Conclusions:**

This is the first population-based study on the prevalence of HCVAb and one of the few population based studies on HBsAg in Zahedan City. We detected lower prevalence rates of HBsAg and HCVAb than in previous studies conducted in Zahedan City. In addition to improvements in social awareness and general health elements, we think that the observed low prevalence rates have been achieved due to the efficiency of mass vaccination projects, implemented against HBV infection in Iran.

## 1. Background

Worldwide, viral hepatitis is one of the leading health problems, accounting for a great number of deaths (1). Hepatitis B virus (HBV) and hepatitis C virus (HCV) infections are two of the most common chronic viral liver diseases (1-3). Hepatocellular carcinoma (HCC) and liver cirrhosis, as chronic sequelaes of viral hepatitis, lead to approximately one million deaths per year (1), while HCV is the number one cause of liver transplantation in the United States and many other countries (4). Moreover, even though HBV vaccination in the majority of countries, including Iran, is expected to decrease the prevalence of this infection, it will take decades to determine the efficiency of these vaccination programs (5). The absence of any HCV vaccine, along with increasing rate of high risk behaviors, raises concerns about the escalating prevalence of HCV infection (6). On the other hand, despite the areas with high or low endemicity with high prevalence rates due to only one particular transmission route, in Iran as a moderate endemicity country, a wide variety of transmission routes are proposed (7). These all highlight the need to carry out studies, especially on the epidemiology of viral hepatitis, in order to establish a better estimate of the burden of disease and subsequent policy making (8).

Many years after the first reports on the prevalence of HBV and HCV, which were published in Iran between 1972 and 1994 (9, 10), conducting similar studies shows an increasing trend in recent years. Most of these studies were carried out on special populations including; hemophilia or thalassemic patients, intravenous drug users and blood donors (11-16). However, sufficient nation-wide population based studies are still lacking (7). On the other hand, Iran is a large country, with both widespread ethnic and cultural diversity, thus the prevalence of HBV and HCV differed significantly in previous reports (17).

Zahedan, the capital city of the Sistan and Baluchestan Province is located in the southeast of Iran, near the border between Iran and Pakistan. This region is populated by people with distinct cultural characteristics, which differ from other parts of the country. Previous studies on the prevalence of viral hepatitis in Zahedan City have been mostly conducted on hemophilic or thalassemic patients, blood donors, pregnant women, and barbers and these have reported various, and sometimes considerably higher prevalence rates: 0.3% to 31.4% for hepatitis B surface antigen (HBsAg), and 13.5 to 29.6% for anti-hepatitis C virus antibody (HCVAb) (15, 18-21). However, extending the results from these studies on high risk subjects to the wider general population in Zahedan City cannot be justified. There has only been one population based study on the prevalence of HBV infection in the Sistan and Baluchestan Province of Iran, which reported a prevalence rate of between 3.38% and 23.58% for HBs Ag and hepatitis B core antibody, respectively (22).

## 2. Objectives

The present study aimed to identify the population-based prevalence of HBsAg and HCVAb and their associated factors in Zahedan City, Iran.

## 3. Patients and Methods

### 3.1. Study Population and Sample Design

Using the random, cluster sampling approach, this cross-sectional study was carried out on 1 380 males and 1 207 females in Zahedan City, Iran during 2008 to 2009. At the time of the implementation of this study, the total number of people living in Zahedan City, according to the latest national capitation survey was 487 031. The study population was comprised of individuals over 10 years of age. The Family Registry at public health centers (n = 40) were considered as a sampling frame. Each public health center covers a separate region of the city. To obtain a representative sample of the civilian population, two clusters with an average size of 30 individuals, were selected from each public health center registry using computer generated random numbers. Two trained interviewers then visited subjects’ homes and provided them with information about the study and its goals. Individuals who met the eligibility criteria were recruited after informed consent was obtained. Subjects who did not give consent, or those who were not available after two contact attempts had been made, were excluded from the study and replaced with the next random subjects. Using this approach, a total of 80 clusters with 2 587 individuals were included in this survey.

### 3.2. Data Collection, Blood Sampling and Laboratory Testing

Participants were interviewed in their homes, and a questionnaire on personal information was completed by a trained interviewer, for each subject. Participants were then asked to refer to the Health Promoting Research Center, and they were provided with an introduction letter for blood sampling. One day after the interview, a 10 mL sample of venous blood was collected into ethylene di-amine tetra-acetic acid (EDTA) bottles, after tourniquet application at the Health Promoting Research Center and then transferred to the regional laboratory of the Iranian Blood Transfusion Organization (IBTO).

Blood samples underwent qualitative evaluations to assess any necessity for repeated blood sampling. Sera were separated for HBsAg and HCVAb assay by centrifugation. Serum samples were then tested for the presence of HBsAg and HCVAb by standard enzymatic kits (Enzygnost, Siemens, Germany and Hepanostika, Beijing United Biomedical, China, respectively) and ELISA Freedom Evolyzer (TCAN, Switzerland). Two copies of the laboratory test results were prepared. One of these was delivered to the participants and another was encoded and an anonymous copy was kept for the study.

### 3.3. Ethics

The study was approved by the Ethical Committee of the Zahedan University of Medical Sciences. Written informed consent was obtained from all of the participants and personal data were kept confidential both during and after the study. The results of the viral marker tests were given to each of the participants (over 18 years old) or their parents (for participants under 18 years old).

### 3.4. Statistical Analysis

Data were analyzed using SPSS software (version 16, IBM ©, USA). Prevalence estimates were weighted to account for non-responsiveness of the study subjects, with weights equal for male and female populations, and also equal for populations in different age groups. Frequency tables were used to calculate the prevalence of HBsAg and HCVAb. The proportional relationship was evaluated by a Pearson’s chi-square test and the linear association between age and viral hepatitis markers was assessed by a chi-square test for trends. In univariate regression analysis, P value < 0.2 was considered as a threshold level of significance to include the variable into the multivariable regression model. Odds ratio for each potential risk factor in a multivariate logistic regression model was used to assess the adjusted impact of these factors on the prevalence of viral hepatitis markers. For categorical variables with more than two layers, dummy variables were created in a logistic regression model and the first layer of the variable was considered as the reference value. For all the other analyses a P value < 0.05 was considered statistically significant.

## 4. Results

A total of 2 587 individuals with mean age of 36.6 (confidence interval 95%: 35.9-37.3, range: 10-88) years old were included in this study. Males constituted 53.3% of the subjects. Of those subjects who were invited to participate in the study, 2.4% were excluded and consequently were substituted with other random subjects. The most prevalent reasons for exclusion were reluctance to participation or non-availability of subjects during contact attempts. Baseline characteristics of the participants are displayed in Table 1.

**Table 1:** Baseline Characteristics of Participants

	**No. (%)**
**Age, y**	
< 18	379 (14.7)
18-25	403 (15.6)
25-35	470 (18.2)
35-45	443 (17.1)
45-55	456 (17.6)
55-65	277 (10.7)
> 65	156 (6)
**Gender**	
Male	1 380 (53.3)
Female	1 207 (46.7)
Married	1 810 (70)
**Race**	
Fars	631 (24.4)
Sistani and Baluchi	1 861 (71.9)
Other	95 (3.7)
Smoking	97 (3.7)
Alcohol usage	10 (0.4)
**Education **	
Less than 12 years	1 943 (75.1)
More than 12 years	644 (24.9)
**Job **	
Unemployed	240 (9.3)
Worker	111 (4.3)
Driver	57 (2.2)
Student	518 (20)
Other	1 661 (64.2)

**Figure 1:**
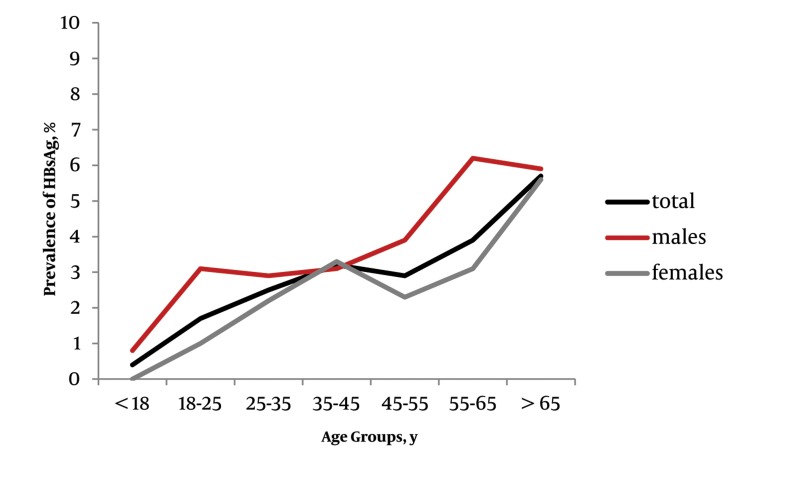
Age-Specific Prevalence of Hepatitis B Surface Antigens

**Figure 2:**
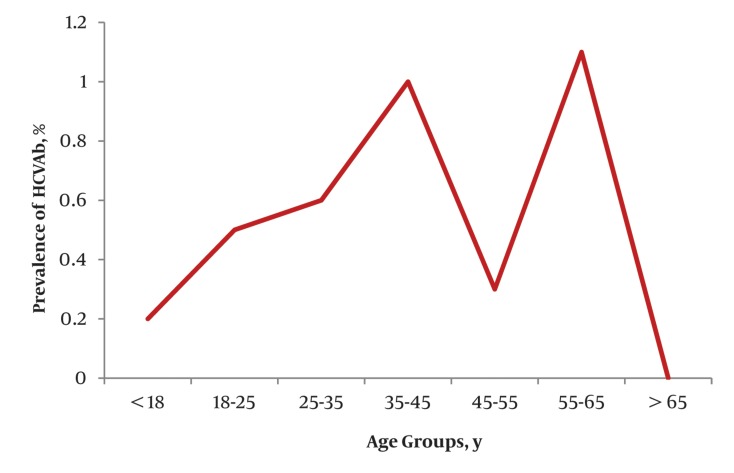
Total Age-Specific Prevalence of Anti-Hepatitis C Virus Antibodies

The overall weighted seroprevalence of HBsAg and HCVAb were 2.5% (CI 95% : 1.9 to 3.3%) and 0.5% (CI 95% : 0.27 to 0.9%), respectively. Although the crude seroprevalence of HBsAg was higher in the males (3.2%) than females (2.2%), however, this difference did not reach statistical significance in proportional analysis (P value, 0.15, Table 2). Moreover, we did not find any significant gender preponderance in the prevalence of HCVAb (P value, 0.273, Table 2) between males (0.4%) and females (0.7%). Table 3 shows the age-specified seroprevalence of HBsAg and HCVAb. Overall and gender specified crude prevalence of HBsAg were linearly associated with age groups (Table 2 and 3, Figure 1). The highest prevalence of HBsAg was observed among subjects over 65 years old. Regarding HCVAb, we did not detect any significant difference among the separate age groups (Table 2 and 3, Figure 2). Due to the low prevalence of HCVAb, we did not perform any age-specified prevalence analysis in separate male and female groups. The prevalence of concomitant HBsAg and HCVAb seropositivity was zero.

**Table 2:** Chi-Square Test for Differences in Prevalence by Potential Risk Factors

	**HBsAg**		**HCVAb**	
	***P*value for Pearson Chi Square**	***P*value for Chi Square for Trend**	***P*value for Pearson Chi Square**	***P*value for Chi Square for Trend**
**Age group**	0.151	-	0.273	-
Male	0.114	0.004	0.646	0.876
Female	0.145	0.007	0.806	0.570
Total	0.013	> 0.001	0.513	0.674
**Race **	0.268	-	0.772	-
**Marital status**	0.001	-	0.59	-
**Literacy level**	0.926	-	0.164	-
**Past surgical history**	0.798	-	0.438	-
**Smoking**	0.661	-	0.473	-
**Alcohol use **	0.593	-	0.821	-
**Job status **	0.076	-	0.389	-

Abbreviations: HBsAg, hepatitis B surface antigen; HCVAb, hepatitis C virus antibody.

**Table 3:** Age-Specified Prevalence of Hepatitis B Surface Antigens and Anti-Hepatitis C Virus Antibodies

**Age Group, y**	**Total Prevalence, %**		**Prevalence, Male, %**		**Prevalence, Female, %**	
	**HBsAg**	**HCVAb**	**HBsAg**	**Anti HCVAb**	**HBsAg**	**Anti HCVAb**
**< 18**	0.4	0.2	0.8	0.4	0	0
**18-25**	1.7	0.5	3.1	0.5	1	0.5
**25-35**	2.5	0.6	2.9	0	2.2	0.9
**35-45**	3.2	1	3.1	0.8	3.3	1.1
**45-55**	2.9	0.3	3.9	0	2.3	0.5
**55-65**	3.9	1.1	6.2	0.9	3.1	1.2
**> 65**	5.7	0	5.9	0	5.6	0

Abbreviations: HBsAg, hepatitis B surface antigen; HCVAb, hepatitis C virus antibody.

As shown in Table 2, there was no significant difference in the prevalence of HCVAb between layers of the potential risk factors. Furthermore, we did not detect a P value < 0.2 for potential predictors of HCVAb in univariate regression analysis (Table 4) and consequently did not perform multivariate logistic regression analysis. The prevalence of HBsAg differed significantly by age group and marital status (with a higher prevalence in married individuals), but not according to gender, race, educational years (less than 12 years vs. 12 years and over), job status, past history of surgery, smoking or alcohol use (Table 2). In univariate regression analysis the prevalence of HBsAg was associated with; age, gender and marital status with P values < 0.2. Moreover, as race and job status had more than two layers, Fars race and unemployment were considered as reference layers for these variables respectively, to be compared with the remaining layers. The latter two variables were also related to HBsAg prevalence with P value < 0.2 in univariate regression analysis and were included in a multivariate model. We were not able to perform a univariate analysis for alcohol usage for HBsAg and HCVAb and also for smoking regarding HCVAb, due to the presence of empty cells (Table 4).

**Table 4:** Risk Factors Associated With Hepatitis B Surface Antigens and Hepatitis C Virus Antibody Seropositivity in Univariate and Multivariate Logistic Regression Analysis

	**HBsAg, Univariate Analysis**			**HBsAg, Multivariate Analysis**			**HCVAb, Univariate Analysis**		
	**Odds Ratio**	**Confidence Interval (95%)**	***P *value**	**Odds Ratio**	**Confidence Interval (95%)**	***P *value**	**Odds Ratio**	**Confidence Interval (95%)**	***P *value**
**Age, y**	1.03	1.01-1.04	< 0.001	1.02	1-1.03	0.043	1	0.97-1.04	0.698
**Gender (F/M) **	0.7	0.43-1.14	0.153	0.72	0.42-1.23	0.229	1.8	0.6-5.7	0.280
**Marital status (M/S)**	3	1.49-6.08	0.002	3	0.93-9.68	0.065	1.42	0.4-5.2	0.592
**Race compared to Fars race as baseline**									
Sistani and Baluchi	1.06	0.6-1.87	0.842	1.22	0.67-2.22	0.510	1.13	0.31-4.11	0.854
Other	2.2	0.79-6.2	0.130	1.74	0.58-5.2	0.323	-	-	-
**Past surgical history**	1.16	0.36-3.8	0.798	-	-	-	2.2	0.28-17.13	0.45
**Smoking**	0.73	0.17-3.01	0.662	-	-	-	-	-	-
**Alcohol usage**	-	-	-	-	-	-	-	-	-
**More than 12 years *vs.* less than 12 years of education **	1.02	0.59-1.79	0.926	-	-	-	0.26	0.03-2	0.207
**Job status compared to unemployed as baseline**									
Worker	1.1	0.32-3.65	0.908	0.8	0.23-2.8	0.725	4.35	039-48.5	0.232
Driver	2.2	0.64-7.6	0.209	2.02	0.56-7.33	0.282	-	-	-
Student	0.4	0.14-1.11	0.079	1.54	0.39-6.12	0.534	0.93	0.08-10.3	0.952
Other	0.85	0.4-1.8	0.686	0.87	0.37-2.05	0.762	1.17	0.14-9.4	0.882

Abbreviations: HBsAg, hepatitis B surface antigen; HCVAb, hepatitis C virus antibody; F/M, female/male; M/S, married/single.

The odds ratios and P values for potential risk factors of HBV infection in multivariate model are outlined in Table 4. The odds of HBsAg seropositivity in married individuals was three times more than in singles, with a P value reaching toward significance (P: 0.065) given that all other variables in the model were held constant. Furthermore, a one year increase in age resulted in a 2% increment in the odds of HBsAg seropositivity, when adjusted for the effects of other variables. Gender, race and job status were not risk factors for HBsAg seropositivity in a multivariate analysis.

## 5. Discussion

In the current study, we observed an overall prevalence rate of 2.5% for HBsAg and 0.5% for HCVAb. On the other hand, in a very recent study, Salehi et al. reported a 3.38% prevalence of HBsAg in the southeast of Iran (22). However, their study consisted of a mixed sample of rural and urban areas, and the higher seroprevalence of HBsAg in rural areas might contribute to this discrepancy with the present study’s results. Moreover, contrary to our results, another study on the prevalence of viral hepatitis in Zahedan was carried out on hemophilic patients in the Zahedan Hemophilia Center and demonstrated a prevalence of 4.9% and 29.6% for HBsAg and HCVAb respectively (15). Similar previous studies have also reported various and mostly higher prevalence rates than ours in thalassemic patients (0.3% for HBsAg and 13.5% and 14.4% for HCVAb), blood donors (16% for HBsAg), pregnant women (6.5% for HBsAg) or barbers (31.4% for HBsAg) in Zahedan (18-21). Hemophilic or thalassemic patients who take blood derived products are exposed to a considerably higher risk of infection with hepatitis viruses than the normal population, which may partly explain the discrepancy between these studies and ours. Blood donors are also not suitable representatives of the general population, since females constitute only about 10% of blood donors (7). In addition to these explanations, we should emphasize the effects of the HBV mass vaccination project that has been implemented in Iran since 1993, which seems to have successfully lowered the prevalence of HBV infection in recent years. The first reports on the prevalence of HBV infection in Iran were published in 1972 (10). Afterwards, a growing number of studies have focused on this issue indicating its importance, and need for further attention. Nevertheless, most of these studies were performed on specific populations such as hemophilic and thalassemic patients or blood donors which limit their generalizability. The number of population-based studies in this regard is still scarce in Iran (9, 11, 12, 14, 15, 23, 24). On the other hand, reports on prevalence rates of HBsAg in Iran vary widely from 1.2 to 9.7% in the general population (25, 26). In addition to conducting studies on different populations which yields discrepancies in results, the prevalence rates of infection vary widely in the different provinces of Iran (17). For example, a recent meta-analysis on 24 population-based studies in Iran reported a total HBV infection prevalence of 2.14%, however, the weighted prevalence rates in East Azarbaijan, Tehran , Hormozgan and Golestan provinces up to 2005 were 1.3%, 2.2%, 2.4% and 6.3%, respectively (17). Iran is populated by people with diverse ethnicities, cultures and beliefs which might also affect the prevalence rates of viral hepatitis. Accordingly, it might not be appropriate to generalize the results of a local study to other provinces, and thus further studies in the various provinces should be conducted. At the beginning of the current study, we had expected high prevalence rates of HBsAg to occur in Zahedan City, due to the close relationship that residents have to the neighboring country, Pakistan with high prevalence of viral hepatitis. But despite our expectations, the prevalence rate in Zahedan was significantly lower than the Baluchestan Province of Pakistan, which has a prevalence rate of 9.8%. (27). We think this is mostly due to the effects of the mass vaccination project against HBV in Iran.

The mass vaccination of neonates and high risk individuals against HBV (started in 1993), as well as the improvements in social awareness and general health elements, are changing the epidemiology of HBV infection in Iran, along with other parts of the world. A review of the current literature reveals that the prevalence of HBV infection is a decreasing trend in Iran (23). However, further reductions in the prevalence of HBV infection is still expected, since the effects of vaccination are discernible only in people younger than 18 years old, but not in older individuals yet (7). On the other hand, the transmission of HBV is now changing from vertical to horizontal routes (23). Nevertheless, despite the abovementioned statements it seems that HBV infection is going to remain the leading cause of end stage liver disease in the next few decades (28, 29). This highlights the need for continuous large, local and national studies on the epidemiology of HBV to produce and carry out appropriate policies to decrease the burden of the disease.

Population-based epidemiologic studies on HCV are fewer in number than those carried out on HBV in Iran. Therefore, it is of the utmost importance that rates of HCV infection is monitored, not only because it is currently a major cause of cirrhosis, hepatocellular carcinoma and liver transplantation (2, 4), but in the future it will soon emerge as the leading cause of chronic viral liver disease, taking into consideration the expanding coverage of mass HBV vaccination and also the increasing rate of HCV infection (6). There have been several reports on the prevalence of HCV in special populations in Iran which have revealed diverse results. HCV infection prevalence in blood donors was reported to be from 0.25% to 0.13% (9, 30) while it was 11-52% for IV drug users (16) and 11-25% for patients on hemodialysis (31, 32). However, the first population-based study on the prevalence of HCV in Iran, which was carried out by Meraat et al. (6), detected an overall prevalence of 0.5% (6). In contrast to our study, they reported a significantly higher prevalence in men than women (1% vs. 0.1%). As stated about HBV infection, conducting studies in different provinces of Iran might be able to explain a part of this discrepancy. A serial review of the literature from past to present emphasizes that the prevalence of HCV infection in Iran is increasing, and strong evidence on the epidemiology of HCV infection along with appropriate policies are required to control the social, economic and health burden of the disease (6).

To date, several risk factors have been proposed for HBV and HCV infections. Despite previous studies (6), HCV did not correlate with any of these factors in the presenting study. On the other hand, the same does not appear to be true about HBV. In proportional bivariable analysis, the prevalence of HBsAg increased significantly with age. In the multivariate analysis, we also observed that the higher the age of the individual, the greater the odds of being HBsAg seropositive. The other interesting and independent predictor of HBsAg seropositivity in our study was marriage, with an odds ratio of three in married compared to single subjects. Gender, race and job status were not risk factors for HBV infection in multivariate analysis. In accord with our results, Merat et al. also showed that age is an independent predictor of HBsAg seropositivity, however, in their study, educational years were also associated with HBsAg (7). In another prevalence study in Qazvin, marriage, lower educational levels, extramarital sexual contact, history of sexually transmitted disease, and close contact with a HBV infected person were also HBV infection risk factors (33).

A possible limitation of the present study might be related to a lack of information about; tattooing, IV drug abuse, high risk sexual contact and similar behaviors, as risk factors of viral hepatitis.

In conclusion, HBV and HCV infections are two of the most serious public health problems all over the world, especially in developing countries (1). We detected lower prevalence rates of HBsAg and HCVAb than in previous studies in Zahedan City. Although the public health projects such as mass vaccination against HBV are changing the epidemiology of viral hepatitis, it would seem that HBV infection will still remain as one of the most important chronic viral diseases for many years to come (28, 29). Furthermore, the increasing rate of high risk behaviors and horizontal transmission of viral disease has resulted in a significant rise in the prevalence of HCV, a finding that suggests that an even more important impact of HCV infection will present itself in the near future. In Iran, like most developing countries, long term studies on the epidemiology of viral hepatitis are still needed in order to address the impact of this serious health issue and its long-term consequences on public health.
